# Associations between class-level factors and student physical activity during physical education lessons in China

**DOI:** 10.1186/s12966-024-01703-6

**Published:** 2025-01-02

**Authors:** Yulan Zhou, Lijuan Wang, Ruzhuan Chen, Bingnan Wang

**Affiliations:** 1 College of Physical Education and Health Sciences, Zhejiang Normal University, Jinhua, 321004 China; 2 School of Physical Education, Shanghai University of Sport, Shanghai, 200438 People’s Republic of China; 3 Physical Education Department, Harbin University of Science and Technology, Rongcheng, 264300 China; 4 Physical Education Department, Central South University, Changsha, 410083 China; 5 School of Physical Education, Shanghai University of Sport, Changhai Road No. 399, Yangpu District, Shanghai, 200438 People’s Republic of China

**Keywords:** Elementary school, Middle school, Physical education, Physical activity

## Abstract

**Background:**

The purpose of this study is to explore the association between class-level factors, such as lesson start time, class size, lesson location, PE content, and PE context, and student engagement in moderate-to-vigorous physical activity (MVPA) during PE lessons in both elementary and middle schools.

**Methods:**

A total of 284 PE lessons from ten schools in Shanghai, Eastern China, were included in the study. Students’ MVPA during PE lessons was recorded using accelerometry, and lesson context was evaluated using the System for Observing Fitness Instruction Time (SOFIT). Mixed linear regression analysis was applied to assess the association between class-level factors and MVPA during elementary and middle school PE lessons.

**Results:**

Students in elementary school spent 40.3 ± 8.1% of PE lesson time in MVPA, while middle school students spent 40.5 ± 7.1%. Significant relationships were found between MVPA and class-level factors like lesson location, PE content, and PE context. Specifically, elementary school students recorded a higher percentage of MVPA during lessons with team games, individual games, individual activities, and more time spent on skill practice and game play context. In middle schools, higher MVPA was connected to outdoor lessons, a focus on individual games, and more time devoted to fitness context.

**Conclusions:**

Class-level factors may affect students’ MVPA differently depending on the school level, and these modifiable factors should be targeted to increase MVPA time in elementary and middle school PE classes. Future studies should investigate ways to modify these factors, strategically plan lesson time across different contexts, and optimizing PE content to boost MVPA in PE lessons.

## Background

School physical education (PE) has been identified as a successful strategy for enhancing physical activity (PA) among children and adolescents [1]. PE offers a structured setting where children and adolescents can participate in PA and acquire motor skills and knowledge that facilitate ongoing engagement in PA [2]. According to guidelines from the United States Department of Health and Human Services (USDHHS) [3] and the United Kingdom Association for Physical Education (afPE) [4], elementary and secondary schools are advised to dedicate at least 50% of their PE class time to moderate-to-vigorous physical activity (MVPA). Despite these recommendations, research conducted by Hollis et al. [5, 6] indicates that students in both elementary and secondary schools typically do not meet this recommended level of engagement.

Given that students’ activity takes place within the context of PE classes, examining class-level variables is an essential step in helping teachers understand how to effectively use PE lessons to increase MVPA time. In their review of studies, the authors identified various class-level correlates affecting students’ MVPA during PE lessons in both elementary and secondary schools. These factors included descriptive characteristics such as lesson start time, duration, class composition (gender distribution), location, and size (number of students), as well as PE content, typology, and context [7, 8]. Barnett et al. [9] found that Australian elementary school students’ MVPA levels were highest when PE lessons started at 9:00 A.M. and lowest at 1:00 P.M. In both Australia and the USA, extended PE lesson durations were correlated with lower levels of MVPA among elementary and secondary school students [10, 11]. Research conducted in the USA indicates that outdoor PE lessons were typically associated with higher levels of MVPA than indoor lessons in both elementary and secondary schools [11, 12]. Two UK studies on secondary school class composition, one found that girls achieved higher MVPA levels in single-gender environments [13], whereas the other found no significant link between class composition and MVPA [14]. Studies conducted in Japan and the USA have reported mixed findings on the association between class size and students’ MVPA. Tanaka et al. [15] indicated that larger class size was associated with reduced MVPA time among elementary school students, whereas Gill et al. [12] observed no significant relationship in secondary schools.

PE content (refers to the focus of a PE class), classified into four types including team games, individual games, movement activities, and individual activities [16], significantly impacted students’ MVPA levels. Research consistently showed that students achieved the highest MVPA levels during team games [16–19], while MVPA levels were typically lowest during movement activities [15, 16, 19] based on studies conducted in the UK, USA, Spain, and Finland across both elementary and secondary schools. As for PE class typology, the study found that Portuguese elementary and secondary school students achieved higher MVPA levels in polythematic PE classes, which included a variety of sports and exercises, compared to monothematic PE classes focused on one sport throughout the session [20].

The PE context, which refers to the various tasks students engage in during PE classes, has been shown to be a significant factor influencing MVPA levels. The System for Observing Fitness Instruction Time (SOFIT) is used to observe this context, with categories such as management (e.g., time devoted to class business unrelated to instructional activity, such as taking attendance, transitions, and breaks), knowledge (e.g., time focused on teaching PE-related knowledge rather than PA), fitness (e.g., activities targeting cardiovascular endurance, strength, or flexibility), skill practice (e.g., developing skills through practice), game play (e.g., time spent in games or competitive activities), and free play (e.g., unstructured playtime not intended for formal PE instruction) [21]. Studies have shown that the PE contexts of fitness, game play, and skill practice were associated with higher MVPA levels in elementary school PE classes in the UK, Australia, and Mexico [9, 22, 23]. In contrast, the PE context of knowledge has been linked to lower MVPA levels in middle school PE classes in the USA and Australia [10, 24].

With changes in school level, students’ MVPA during PE classes also varies. Meta-analyses by Hollis and colleagues on MVPA during PE lessons found that the percentage of MVPA was higher in secondary school PE classes (48.6%) compared to elementary school PE classes (44.8%) [5, 6]. Meanwhile, Metzler [25] proposed that with shifts in school level, teachers adjust their management of the various components of a lesson and select activities that align with students’ developmental stages to foster an appropriate learning environment. Therefore, the association between class-level factors and students’ PA behaviors may differ across school levels. Recognizing these potential differences in how these factors influence students’ MVPA across school levels can assist in devising targeted and effective interventions. However, only one study by Kwon et al. [26] has examined class-level factors influencing students’ MVPA across elementary, middle, and high school PE settings. The study found that the fitness context was positively associated with MVPA across all school levels, while game play context was significantly linked to MVPA only in elementary PE. Additionally, students engaged in significantly more MVPA during outdoor lessons compared to indoor lessons in elementary and middle school PE, but no significant difference was observed in high school PE. Nevertheless, evidence on the differences in the association between class-level factors and students’ MVPA time during PE across different school levels remains limited, highlighting the need for further research to confirm these variations.

In China, PE is mandatory for all students from grades 1 through 12, as well as at the university level. The Curriculum Standard for physical education and health compulsory education, set by the Chinese Ministry of Education, outlines guidelines for PE teaching in elementary and middle schools, covering curriculum objectives, content, and assessment systems. The elementary and middle school PE curriculum in China aims to develop students’ motor competence, including mastering fundamental motor skills, physical fitness, and sport-specific skills. It promotes healthy behaviors through good exercise, diet, rest, and hygiene habits. Moreover, the curriculum aims to nurture sports ethics, emphasizing the code of conduct in physical activities and fostering values and a positive spiritual outlook [27]. Schools and teachers have the autonomy to select PE content, such as sports skills, games, and fitness routines, and determine how lesson are delivered to meet these educational aims [27]. The Curriculum Standard emphasized the importance of ensuring appropriate MVPA time in PE classes to enhance students’ motor competence and healthy behaviors. The extent to which students are physically active is a significant factor in evaluating PE class quality. Research has explored how teacher-related factors (e.g., teacher characteristics, teaching behavior) [28, 29], environmental conditions (e.g., class location, size of activity area) [30, 31], and psychological aspects of students (e.g., basic psychological needs, motivation levels) [32] influence the MVPA levels of Chinese PE students. However, no study has specifically examined the connection between PE class-level factors and students’ MVPA in China.

Based on these research gaps, this study aimed to examine the association between class-level factors and students’ MVPA in PE lessons across elementary and middle schools in China. The class-level factors such as PE classroom characteristics (e.g., lesson start time, class size, lesson location), PE content, and PE context are included in the present study based on previous studies [14, 18]. Variables such as lesson duration, class composition, and class typology are not applicable to this study, as these elements are consistent across elementary and middle school PE classes in China, which follow standardized formats of co-educational, polythematic lessons. The findings are intended to assist school administrators and physical educators in optimizing lesson structures and designing targeted interventions aimed at increasing students’ MVPA during PE lessons in these school settings.

## Methods

### Study design

This cross-sectional study adhered to the Strengthening the Reporting of Observational Studies in Epidemiology (STROBE) checklist for reporting observational studies [33] (see Additional file 1).

### Participants and PE setting

The research procedures were approval by the Ethics Committee of Zhejiang Normal University and the relevant school authorities. The study was conducted in five elementary and five middle schools, chosen from four districts in Shanghai, East China. Schools were selected according to the following inclusion criteria: (a) public schools were chosen from four Shanghai districts representing diverse socioeconomic statuses; (b) school principals agreed to participate; (c) each school needed to offer at least four classes per grade; (d) the PE facilities and equipment at each school were assessed to ensure comparability. The schools were considered representative of the elementary and middle schools in Shanghai based on their size, type, and location within each district. Finally, the ten schools included in this study have four to eight intact classes per grade in elementary school (Grades 1 to 5) and six to seventeen intact classes per grade in middle school (Grades 6 to 9). From each grade level at these elementary and middle schools, three to four classes were randomly selected. Every student within these selected classes received an invitation to participate in the study. A total of 4,634 students from 143 classes across elementary and middle schools were invited, with 4,482 students (96.7%) ultimately agreeing to participate.

Following the Curriculum Standard guidelines, elementary and middle schools are mandated to deliver four weekly PE lessons for Grades 1 and 2, and three weekly lessons for Grades 3 to 9. PE lessons in elementary and middle schools are co-educational, with roughly equal numbers of males and females, and lasted 35 and 40 min, respectively. Elementary and middle schools follow a schedule from Monday to Friday, from 8:00 A.M. to 5:00 P.M. PE lessons are conducted in various environments, including indoor facilities like gymnasiums and outdoor settings such as school fields and basketball courts. All PE instruction is provided by specialist teachers with a bachelor’s or master’s degree in PE. In a standard PE class, there are three main parts: a 5-minute warm-up and introduction led by the PE teacher; a 25-30-minute segment where the teacher demonstrates and explains motor or sport skills, then organizes students for skill practice; and finally, a 5-minute conclusion led by the teacher.

### Variables and measures

#### PE classroom characteristics

Descriptive characteristics of the PE classroom were recorded by researchers, encompassing lesson start time, class size, and lesson location. Researchers defined lesson start times as either A.M. (before 12:00 o’clock) or P.M. (after 12:00 o’clock). Class sizes in the study area typically ranged from 25 to 40 students. Drawing on prior research from a Chinese context, which identified 21–30 students as the optimal range for evaluating class size effects on performance, this study established 30 as the threshold [34]. Additionally, lesson locations were classified as either “Indoors” or “Outdoors”.

#### PE content

PE content was classified into four types based on activity characteristics, as defined by Fairclough et al. [16]: team games (e.g., football and basketball), individual games (e.g., badminton, table tennis, and volleyball), movement activities (e.g., dance, gymnastics, and martial arts), and individual activities (e.g., athletics, fitness, and swimming). In polythematic classes, the PE content recorded was determined by the activity type that predominated during the majority of the lesson.

#### PE context

SOFIT, recognized for its validity and reliability, was employed to assess the context of PE classes [11]. The tool divides the PE context into various components: management, knowledge, fitness, skill practice, game play, and free play. The context is determined by how time is allocated across the class, with a minimum of 51% student participation [21].

#### Students’ MVPA

Actigraph wGT3X-BT accelerometers were utilized in this study to measure MVPA among students. These devices are recognized for their validity and reliability in youth populations [35]. Longer epochs can underestimate vigorous PA levels in children [36]. Furthermore, because classes are structured in bouts of PA, longer epochs may result in an overestimation of MVPA by merging these bouts. To address this, 1-second epochs were employed for accelerometer data collection in this study [37]. After the test, original Actigraph data files were downloaded from the accelerometers and analyzed using ActiLife software version 6.5. Data recorded at or above the moderate physical intensity level, defined as ≥ 2,800 counts per minute, were converted into minutes to represent MVPA during lessons [38]. The percentage of class time spent in MVPA was calculated by dividing the average time all students spent in MVPA by the total duration of the classes.

### Data collection

Data collection spanned from September 2022 to January 2023, led by the primary author and five graduate student research assistants specializing in Sports Pedagogy. To address seasonal and curricular diversity, observation days across the 10 schools were structured into two 10-week cycles (Fall and Winter), ensuring each class was observed once per cycle. Over the course of five months, whenever cancellations occurred due to factors like inclement weather or school activities such as mid-term exams or sporting events, the subsequent scheduled lesson was selected for observation. This approach yielded a total of 286 intact lessons scheduled for observation (i.e., 143 classes × 2). However, two classes were observed only once due to school-wide flu incidence leading to cancellations. Consequently, the analytic sample consisted of 284 PE classes. Teachers were instructed to maintain typical class activities without testing or unusual activities.

The primary author and five research assistants simultaneously used accelerometers to evaluate students’ MVPA, alongside SOFIT for assessing the PE context during PE classes. Data collection initiated upon the commencement of each class and concluded at its termination. At the beginning of each PE class, accelerometers were handed out to all students who signed informed consent to participate in the study. A Sony HDR-XR500 video camera was used to record the classes, strategically positioned in places like the seating area to document the instructional setting. During PE classes, students attached accelerometers to their right hipbones using elastic belts and wore them without interruption. A research assistant ensured correct positioning and monitored students to prevent any removal of the accelerometers. Consequently, the valid wear period for each accelerometer was defined as continuous use throughout the entirety of the PE class. Data were excluded for non-compliance with accelerometer wear resulting from student absence, tardiness, or injury during the PE class.

Research assistants collected PE context data by recording at 20-second intervals to quantify the time students spent engaged in various tasks during classes. This involved alternating between 10 s of direct observation and 10 s of recording. The SOFIT protocol recommends verifying interobserver reliabilities in 12% of all observed lessons [39]. During a five-month observation period, 35 lessons (12.3% of the total) were randomly sampled for reliability checks. These consisted of 15 lessons observed in weeks 1–7 of assessments, 15 lessons in weeks 8–14, and 5 lessons in weeks 15–20. The lessons were independently coded by the primary author and a research assistant, resulting in an interobserver reliability agreement of 84.1% for the PE context, exceeding the 80% threshold recommended by van der Mars [40]. To determine the percentage of class time dedicated to each PE context, intervals where each context occurred were divided by the total observation intervals for the entire class, and the quotient was multiplied by 100.

After each class, research assistants documented lesson start times, class sizes, lesson locations, and PE lesson content using a log sheet.

### Data analysis

All analyses were conducted using SPSS statistical analysis software (v. 26.0). Descriptive statistics were computed for categorical variables (i.e., lesson start time, class size, lesson location, and PE content) using proportions, and for continuous variables (i.e., PE context and MVPA), means and standard deviations were calculated. Differences in class-level factors between elementary and middle schools were measured using Chi-square tests, T-tests, and multivariate analysis of variance (MANOVA). For significant MANOVAs, follow-up one-way analyses of variance (ANOVA) and Least Significant Difference (LSD) post hoc tests were performed to explore differences across school levels. The percentage of MVPA among groups within categorical class-level factors in elementary and middle schools was analyzed using ANOVA. Partial eta-squared (η²) was used as the effect size indicator, with thresholds of 0.01, 0.06, and 0.14 representing small, medium, and large effects, respectively [41]. Given the data structure (lessons nested within schools), mixed linear regression analysis was used to assess the relationship between each class-level factor and students’ MVPA during PE lessons in elementary and middle schools. All class-level factors were treated as fixed effects, with students clustered within elementary and middle schools as a random effect. Significance levels were set at *P* < 0.05.

## Results

### Description of class-level factors and student MVPA during elementary and middle school

Table 1 presents the descriptive statistics of class-level factors and student MVPA in elementary and middle schools. Overall, 284 PE classes were observed across 10 schools, comprising 138 elementary school lessons and 146 middle school lessons. Of these, 46.4% of elementary PE classes and 52.7% of middle PE classes were conducted in the A.M. More than half of the PE classes in both elementary (65.9%) and middle school (65.1%) had more than 30 students. The majority of PE lessons were held outdoors, with 82.6% in elementary schools and 78.8% in middle schools. In both elementary (47.1%) and middle schools (45.9%), individual activities were the most frequently taught PE content, with substantial portions of class time being used for management (43.1% in elementary schools and 37.3% in middle schools). MVPA levels in elementary (40.3%) and middle school (40.5%) PE classes did not meet the standard of 50% of class time. Chi-square analyses indicated significant differences in PE content between school levels. Elementary school PE classes featured more individual games than middle school classes (*P* < 0.05), while middle school classes included more team games (*P* < 0.05). MANOVA results revealed significant differences in the proportion of PE context between school levels [F (2, 281) = 7.348, *P* < 0.001, η^2^ = 0.338]. Elementary schools spent a greater proportion of lesson time to management (*P* < 0.001, η^2^ = 0.125) and game play (*P* < 0.001, η^2^ = 0.168) compared to middle schools, but spent less time on knowledge (*P* < 0.001; η^2^ = 0.065) and free play contexts (*P* = 0.005; η^2^ = 0.089).

**Table 1:** Characteristics of the class-level factors and students MVPA during elementary and middle school

Variables	Elementary school (*N* = 138)	Middle school (*N* = 146)	Statistical result	
Categorical variables	N (%)	N (%)	χ^2^	P
Lesson start time			1.149	0.284
A. M.	64 (46.4%)	77 (52.7%)		
P. M.	74 (53.6%)	69 (47.3%)		
Class size			0.024	0.877
≤ 30	47 (34.1%)	51 (34.9%)		
> 30	91 (65.9%)	95 (65.1%)		
Lesson location			0.670	0.413
Indoor	24 (17.4%)	31 (21.2%)		
Outdoor	114 (82.6%)	115 (78.8%)		
PE content			16.909	0.001
Team games	20 (14.5%)	44 (30.1%)		< 0.05
Individual games	32 (23.2%)	17 (11.7%)		< 0.05
Movement activities	21 (15.2%)	18 (12.3%)		> 0.05
Individual activities	65 (47.1%)	67 (45.9%)		> 0.05
Continuous variables	Mean (SD)	Mean (SD)	F/t	P
PE context			8.305	< 0.001
Management	43.1 (15.7)	37.3 (18.2)	9.127	< 0.001
Knowledge	7.8 (8.6)	13.6 (7.1)	5.776	< 0.001
Fitness	20.9 (14.2)	22.9 (12.5)	0.008	0.984
Skill practice	17.3 (14.8)	14.6 (19.2)	1.939	0.165
Game play	8.9 (14.8)	2.2 (3.4)	9.536	< 0.001
Free play	2.0 (5.9)	9.4 (16.3)	5.507	0.005
MVPA (%)	40.3 (8.1)	40.5 (7.1)	–0.269	0.788

### Proportion of MVPA for class-level factors during elementary and middle schools

**Fig. 1:**
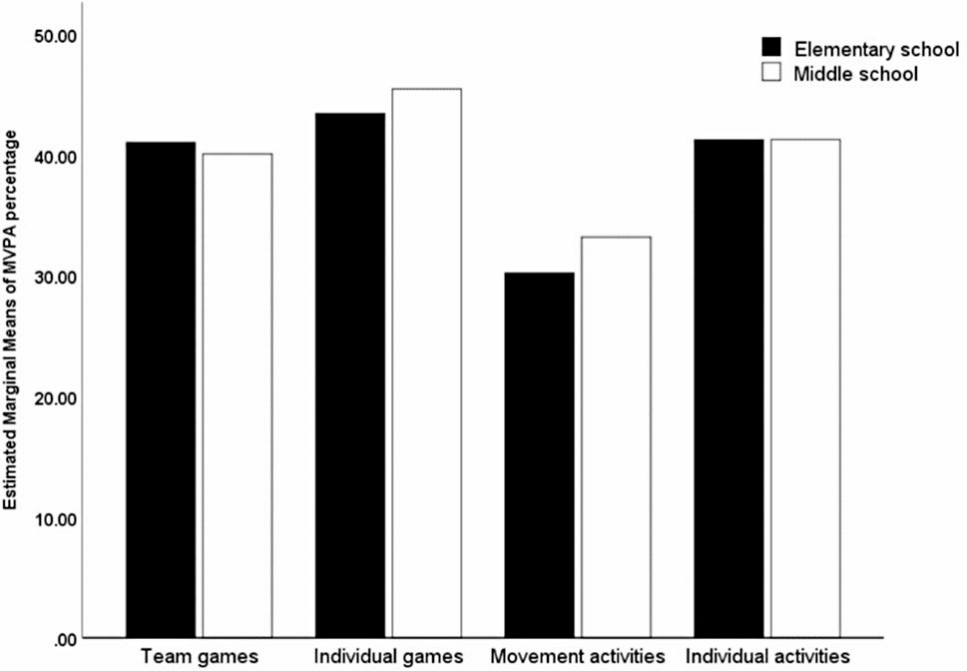
Moderate to vigorous physical activity (MVPA) across different PE contents for both elementary and middle schools

Table 2 displays the differences in MVPA percentages between groups within categorical class-level factors in elementary and middle school. In elementary schools, higher MVPA percentages were observed in PE lesson with a class size of 30 or fewer (*P* = 0.035, η^2^ = 0.032) and in outdoor settings (*P* = 0.001, η^2^ = 0.085). ANOVA results demonstrated significant differences in MVPA time across various PE content types (*P* < 0.001, η^2^ = 0.242). LSD tests indicated higher MVPA percentages during individual games, team games, and individual activities compared to movement activities (*P* < 0.001). However, no significant differences were found between individual games and team games or individual activities. In middle schools, outdoor PE lessons showed higher MVPA percentages compared to indoor lessons (*P* < 0.001, η^2^ = 0.091). Significant differences in MVPA percentages were observed across different PE content categories (*P* < 0.001; η^2^ = 0.170). LSD tests revealed that involving individual games had significantly higher MVPA percentages compared to other content areas (*P* < 0.05). Furthermore, both team games and individual activities exhibited higher MVPA percentage compared to movement activities (*P* < 0.05) (see Fig. 1).

**Table 2:** MVPA percentages between groups within categorical class-level factors in elementary and middle school

	Elementary school (%)				Middle school (%)				
	Mean (SD)	F	P		Mean (SD)		F		P
Lesson start time		0.832	0.363			0.605		0.438	
A.M.	39.6 (8.1)				40.9 (7.8)				
P.M.	40.6 (8.2)				40.0 (6.3)				
Class size		4.516	0.035			0.172		0.679	
≤ 30	42.3 (7.9)				40.2 (7.2)				
> 30	39.2 (8.1)				40.7 (7.0)				
Lesson location		12.581	0.001			14.467		< 0.001	
Indoor	35.1 (7.8)				36.4 (6.2)				
Outdoor	41.3 (7.8)				41.6 (6.9)				
PE content		14.390	< 0.001			9.960		< 0.001	
Team games	41.2 (8.2)				40.1 (6.9)				
Individual games	43.4 (6.8)				45.4 (8.1)				
Movement activities	30.2 (9.6)				33.2 (6.3)				
Individual activities	41.2 (6.2)				41.3 (5.8)				

### Association between class-level factors and students’ MVPA during elementary and middle school PE lessons

The results of the mixed-model regression analysis, detailing the relationship between class-level factors and students’ MVPA time in elementary and middle school PE lessons, are shown in Table 3. In elementary school PE lessons, significant associations were found between PE content, PE context, and students’ MVPA. Lower percentages of MVPA were observed in PE lessons with increased movement activities (β = − 9.230, 95% confidence interval (CI) = − 12.265, − 4.290), more time devoted to management (β = − 2.084, 95% CI = − 3.053, − 0.761) and knowledge (β = − 1.440, 95% CI = − 2.459, − 1.031). Conversely, higher MVPA percentages were associated with more time spent on skill practice (β = 1.618, 95% CI = 0.608, 2.575) and game play (β = 1.397, 95% CI = 0.439, 2.474). Middle school PE lessons showed significant associations between lesson location, PE content, PE context, and students’ MVPA. Lower MVPA percentages were associated with indoor lessons (β = − 3.300, 95% CI = − 5.487, − 1.114), increased time spent on management (β = − 5.739, 95% CI = − 8.109, − 3.369) and knowledge (β = − 1.986, 95% CI = − 3.028, − 0.945). However, PE lessons with more individual activity (β = 5.309, 95% CI = 2.366, 8.253) and greater lesson time devoted to fitness contexts (β = 2.117, 95% CI = 0.840, 3.395) were linked to higher MVPA percentages.

**Table 3:** Mixed-model regression analysis of class-level factors and students’ MVPA during elementary and middle school PE classes

	Elementary school MVPA				Middle school MVPA		
	β	t	*p*		β	t	*p*
Lesson start time- A.M. ^a^	–1.132	–0.942	0.348		0.915	1.059	0.292
Class size- ≤30 ^b^	2.258	1.872	0.064		–0.831	–0.858	0.393
Lesson location-Indoors ^c^	–2.053	–1.186	0.238		–3.300	–2.985	0.003
PE content ^d^							
Individual games	0.418	0.207	0.836		5.309	3.568	0.001
Movement activities	–9.230	–4.422	0.000		–1.843	–1.139	0.257
Individual activities	–0.401	–0.232	0.817		3.076	2.314	0.084
PE context							
Management	–2.084	–1.409	0.016		–5.739	–4.789	< 0.001
Knowledge	–1.440	–2.380	0.019		–1.986	–3.772	< 0.001
Fitness	1.080	1.253	0.213		2.117	3.279	0.001
Skill practice	1.618	3.224	0.002		0.701	1.558	0.122
Game play	1.397	2.716	0.008		–1.009	–1.558	0.122
Free play	1.082	1.627	0.106		–0.033	–0.088	0.930

^a^ Reference category: P. M.; ^b^ Reference category: > 30; ^c^ Reference category: Outdoors; ^d^ Reference category: Team games

## Discussion

According to the present study, elementary and middle school students spent an average of 40.3% and 40.5% of class time in MVPA, respectively. These findings align with previous systematic reviews, which indicated that students engaged in MVPA for 44.8% and 40.5% of PE class time during elementary and middle school, respectively [5, 6]. Current MVPA percentages in PE fall short of the USDHHS [3] and afPE [4] recommendations, which advise that students engage in MVPA for at least 50% of lesson time. PE intervention programs featuring teacher professional development to enhance class organization and management, and the incorporation of high-intensity activities into regular PE content, have proven highly effective, boosting students’ MVPA time by 24% compared to traditional practices [42]. Consequently, there is a need to investigate the factors influencing MVPA among elementary and middle school students during PE classes and to develop intervention strategies to promote MVPA.

### PE classroom characteristics and students’ MVPA

Regression analysis revealed no significant correlation between PE classroom characteristics and students’ MVPA, except for a notable relationship between lesson location and MVPA. The findings showed that middle school students engaged in significantly less MVPA during indoor PE lessons compared to outdoor lessons, whereas no significant relationship was found between lesson location and MVPA among elementary school students. These findings partially align with prior research, which suggests that outdoor PE lessons generally led to higher MVPA levels than indoor lessons in both elementary and secondary schools [11, 12]. Studies indicate that outdoor lessons in natural settings, compared to indoor environments, foster greater feelings of revitalization, positive engagement, increased energy, and decreased tension, confusion, anger, and depression [43]. However, elementary and middle school students may vary in their sensitivity to or perception of these environmental effects. The development of the central nervous system, which supports environmental sensitivity, progresses with age. Middle school represents a critical stage in this process, as increased neural connections may enhance environmental sensitivity in middle school students compared to younger children [44]. This may explain why indoor PE lessons were linked to lower MVPA levels in middle schools but showed no significant effect in elementary schools. These findings underscore the need for strategies to boost MVPA among middle school students during indoor lessons, such as improving facility aesthetics, integrating green spaces, and designing multi-functional activity areas.

### PE content and students’ MVPA

Consistent with previous research [15], the present study found that movement activities had the lowest percentage of MVPA in elementary school PE classes compared to team games, individual games, and individual activities. Regression analysis revealed no significant association between movement activities and MVPA in middle school PE classes. However, ANOVA results showed that MVPA levels during movement activities were significantly lower than in other types of PE content. These findings are expected, as movement-based activities such as dance and gymnastics emphasize aesthetic expression and body control rather than vigorous PA [45]. Unlike other types of PE content that involve simultaneous movement of the arms and legs over varying durations, movement activities often require keeping either the upper or lower limbs immobilized. This can result in a low accelerometer count that fail to reach the cut-off value for MVPA levels [46]. The PE curriculum aims to achieve multiple objectives, including cognitive, physical, moral, and spiritual development. Consequently, it incorporates diverse content, such as movement activities, which often require substantial periods during which students are not physically active.

Middle school students demonstrated significantly higher MVPA levels during individual games in PE classes compared to other content types, such as team games, movement activities, and individual activities. This finding contrasts with earlier studies, which reported the highest percentage of MVPA in team games [16, 19]. Team games, characterized by movements at different speeds, appear to encourage MVPA engagement among students. Nevertheless, these activities require participants to possess adequate skills, tactical knowledge, and motivation [47]. In China, PE classes typically emphasize the acquisition of sports skills, which may result in students having a limited understanding of game strategies and team collaboration within team games [48]. Evidence suggests that a lack of tactical knowledge and awareness presents challenges in engaging Chinese students in team-based activities [49, 50]. In contrast, individual activities such as table tennis, badminton, and volleyball are highly popular in China. Many school students, especially middle schoolers, excel in these sports, making it easier to involve them in such activities [51]. These findings indicate that the PE content delivered by teachers is not the only factor influencing students’ engagement outcomes. A more in-depth analysis of the teaching and learning process is essential to fully leverage the potential of various PE content in achieving educational objectives.

### PE context and students’ MVPA

The management and knowledge components of the PE context were negatively associated with students’ MVPA during elementary and middle school PE classes, consistent with findings from previous studies [10, 52]. These results are expected, as students are often required to observe and listen to the instructor during these contexts rather than actively participate [53]. However, it is important to note that if management and knowledge contexts are in harmony with effective teaching and student motor skill learning, they should be considered necessary elements of PE. To achieve PE objectives and increase time spent in MVPA, teachers should employ thoughtful strategies and carefully plan diverse lesson contexts. This could include engaging students in management tasks, such as setting up equipment, and integrating knowledge delivery with physical movement.

The PE context of game play and skill practice were positively correlated with students’ MVPA in elementary school PE lessons, but did not show a significant association with middle school students’ MVPA time. These findings are consistent with previous studies [22, 23]. Game play is inherently an “active context” in PE, where PA naturally occurs [22]. However, a descriptive analysis of the current study revealed that game play accounted for only 2.2% of middle school lesson time on average. In contrast, elementary school students spent four times as much time in game play (8.9%) compared to middle school students. This greater emphasis on game play significantly influences MVPA levels in elementary school students but appears to have no meaningful effect on MVPA in middle school PE classes. The variation in the effect of skill practice on MVPA between elementary and middle school PE lessons may be attributed to differences in the types of skills taught (e.g., fundamental motor skills such as running, jumping, and throwing versus ball-specific skills like passing, catching, and dribbling) and the instructional methods employed (e.g., incorporating diverse games and competitive scenarios versus structured, repetitive instruction in ball skills) [54]. Further research is needed to investigate and confirm these findings in future studies.

While this study did not find a positive association between the PE context of fitness and elementary school students’ MVPA, as reported in earlier research [22, 23], it did identify such an association in middle school students, consistent with findings from other studies [24, 55]. The fitness context refers to lesson time devoted to activities aimed at improving flexibility, muscle strength, cardiovascular endurance, and muscular endurance. Middle school students, compared to elementary school students, demonstrate greater development in cardiorespiratory endurance, muscle endurance, and muscle strength. As a result, they are more likely to engage in higher levels of MVPA within fitness contexts, as their enhanced fitness allows for sustained participation in higher-intensity and competitive activities [56]. This result highlights that, to enhance MVPA levels in middle school PE lessons, teachers can appropriately extend the time allocated in fitness context and design enjoyable, motivating activity environments.

### Strengths and limitations

This study is one of the few studies to examine the association between class-related factors and students’ MVPA during PE lessons in China. The findings provide valuable insights that could inform interventions aimed at enhancing PA levels among Chinese children. However, certain limitations of this study must be acknowledged. The primary limitation relates to the generalization of its findings. As the participants were draw from ten elementary and middle schools in Shanghai, the results may not fully represent children from other regions of China. Future studies should expand to include a larger and more diverse sample from various regions across the country. Second, the cross-sectional nature of the data limits the ability to draw causal inferences about the relationships between class-level factors and students’ MVPA during PE lessons. Longitudinal and intervention-based studies are needed to establish causal links between these factors. Third, unmeasured confounding factors may have biased our results. Future studies should consider including additional variables, such as class composition and students’ motivation in PE. Lastly, the potential for reactive behavior from both students and teachers due to the presence of researchers observing the lessons should be acknowledged. Seamlessly integrating observation into everyday teaching practices may help mitigate this effect.

## Conclusions

In conclusion, this study highlighted class-level factors influencing the percentage of MVPA during PE lessons across school levels. For elementary students, participating in team games, individual games, individual activities, and dedicating more time to skill practice and game play in the PE context are likely to boost MVPA. In middle school, higher MVPA levels are associated with outdoor lessons, individual games as the primary activity, and increased time spent in fitness PE context. School administrators and PE educators can focus on the modifiable factors identified in this study to implement interventions aimed at increasing MVPA time in elementary and middle school PE lessons.

## Data Availability

The datasets used and/or analyzed during the current study are available from the corresponding author on reasonable request.

## Abbreviations

PE: Physical Education
MVPA: Moderate-to-Vigorous Physical Activity
USDHHS: US Department of Health and Human Services
afPE: Association for Physical Education
SOFIT: System for Observing Fitness Instruction Time
STROBE: Strengthening the reporting of observational studies in epidemiology
MANOVA: Multivariate Analysis of Variance
ANOVA: Analysis of Variance
LSD: Least Significant Difference
CI: Confidence Interval
